# HASTE MRI in the assessment of children with hydrocephalus

**DOI:** 10.1186/1743-8454-7-S1-S36

**Published:** 2010-12-15

**Authors:** Samuel R Browd, Brent R O’Neill, Sumit Pruthi, Harmanjeet Bains, Ryan Robison, Keiko Weir, Jeff Ojemann, Anthony Avellino, Richard Ellenbogen

**Affiliations:** 1 Department of Neurological Surgery Seattle Children's Hospital PO Box 5371, Seattle, WA 98105-0371, USA

## Background

Recent reports and our clinical experience have shown the usefulness of rapid-acquisition MRI in evaluating children with hydrocephalus. An axial HASTE (i.e. Half-Fourier Acquisition Single-shot Turbo Spin Echo Magnetic Resonance Imaging) MRI study acquires clinically useful images in seconds without exposing children to the risks of ionizing radiation or sedation. The current report reviews the Seattle Children’s Hospital experience with rapid acquisition MRI in shunted children with attention to ventricular size, overall image quality, motion artifact, and catheter visualization.

## Materials and methods

All HASTE MRIs obtained at Seattle Children’s Hospital over two-years were reviewed by two evaluators on scales of overall image quality, catheter visualization, motion artifact, and ventricular size. Relationships among these factors were sought.

## Results

Overall image quality was rated very good or excellent in 94% of the studies reviewed, while only one study was graded as poor. Significant motion artifact was noted in 7% while 77% had little or no motion artifact. Catheter visualization was rated as good or excellent in 57% of studies reviewed, poor in 36%, and misleading in 7%. Small ventricular size showed a significant correlation with poor catheter visualization (Pearson correlation coefficient = .575; p<.00001). Ventricular enlargement concerning for shunt malfunction on HASTE imaging correlated with operative findings of shunt malfunction in 100% of cases taken to the OR based on HASTE imaging.

## Conclusions

Our study adds further support to the emerging evidence that HASTE MRI is an adequate substitute for CT scanning allowing for reduced utilization of CT imaging and resultant exposure to ionizing radiation. Visualization of catheter position remains suboptimal with HASTE MRI, particularly when ventricles are small; however, shunt malfunction can be adequately determined based on ventricular size alone in the majority of cases.

